# Synchronous Multiple Primary Cancers of the Lung: The Rare Association of Non-Small Cell Carcinoma With a Carcinoid Tumor

**DOI:** 10.7759/cureus.9888

**Published:** 2020-08-20

**Authors:** Meghana Parsi, Raymond J Vivacqua

**Affiliations:** 1 Internal Medicine, Crozer-Chester Medical Center, Upland, USA; 2 Hematology and Medical Oncology, Crozer-Chester Medical Center, Upland, USA

**Keywords:** carcinoid tumor, metastatic non-small cell lung cancer, synchronous tumor

## Abstract

A 68-year-old female patient diagnosed with lung cancer in the left upper lobe with associated mediastinal adenopathy. The cancer was pathologically diagnosed as stage pT1bN0 typical carcinoid. Investigation of the mediastinal lymph nodes revealed an isolated metastatic non-small cell squamous cell carcinoma (NSCLC). A primary NSCLC was not found. The patient underwent successful surgical resection of both synchronous tumors, with no residual disease or recurrence. This case not only expands the histological field of combined neuroendocrine tumors, but it also highlights the importance of distinguishing various tumor types for disease treatment and prognosis.

## Introduction

Despite recent advances in technology, lung cancer remains the leading cause of cancer-related deaths. Multiple primary lung cancers, either synchronous or metachronous, are unusual. Synchronous tumors are those in which pathologically and histologically different tumors exist at the same time. Cases of synchronous but discordant histology with small cell and non-small cell lung carcinoma (NSCLC) occurring in the same patient are rarely reported. To date, there are only a few reported cases of synchronous lung tumors consisting of both NSCLC and carcinoid tumors in the medical literature [1]. Metachronous tumors are tumors that are also histologically different from one another but occur at different times in the patient. Diagnosis will pose a challenge as the presence of multiple tumors at the same time can be mistaken to be metastatic lesions. Knowing the histological types of these various tumors can dictate management and have prognostic implications. We report the clinical and pathological findings of a case of a combined typical carcinoid and squamous cell carcinoma of the lung. In the age of advanced molecular, genetic, and radiology techniques, differentiation between different tumor types is important to tailor appropriate treatment modalities.

## Case presentation

**Figure 1:**
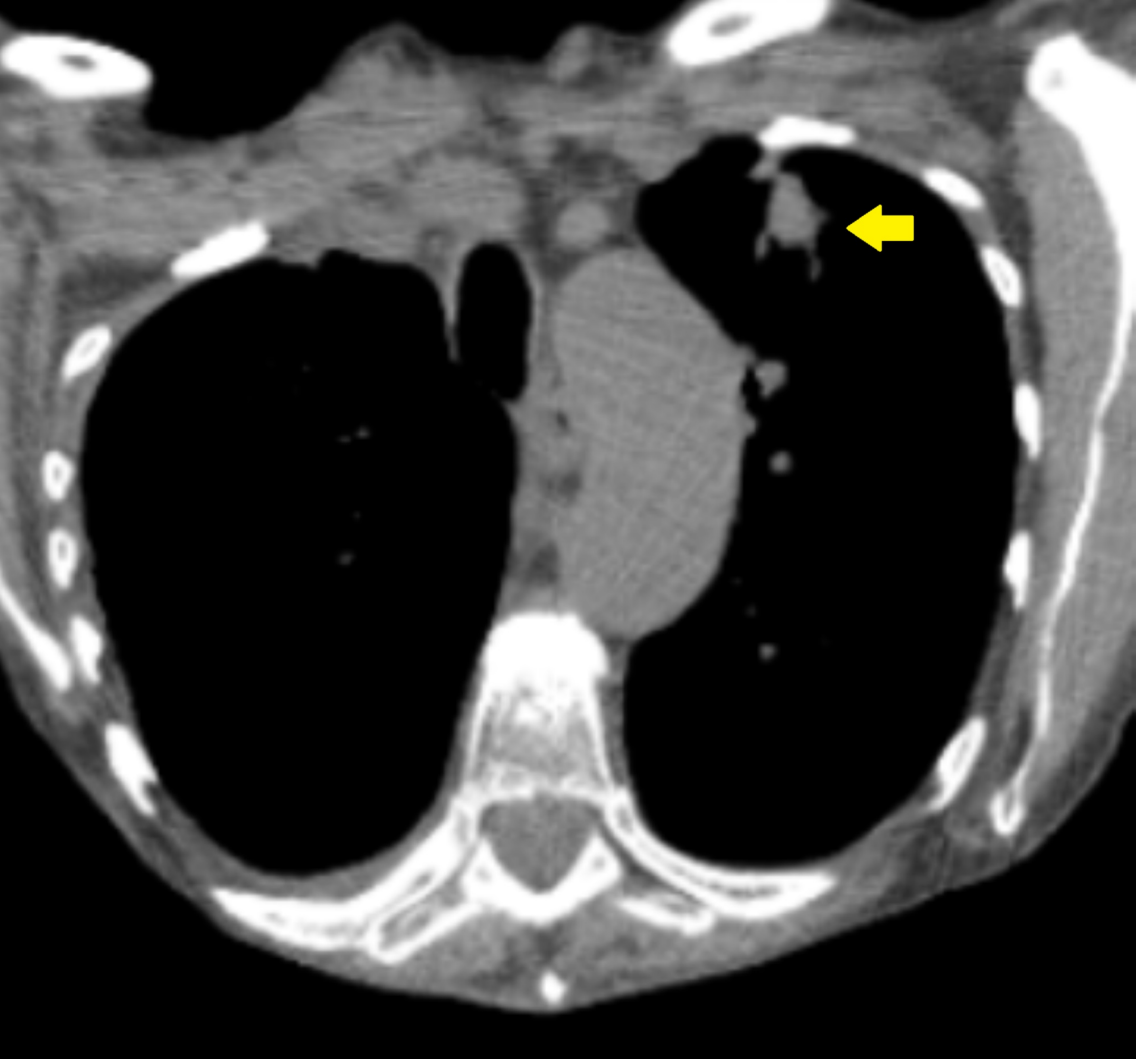
CT of the chest The yellow arrow pointing towards a lesion in the left upper lobe of the lung that is approximately 2.8-3.0 cm in diameter.

A 68-year-old female patient presented after an abnormal lung shadow was found during a routine radiological assessment. The patient has a prior history of hypertension, hyperlipidemia, and patent foramen ovale. She was a former smoker with a 45 pack-year history. A follow-up chest CT for the abnormal shadow revealed a left upper lobe nodule measuring 2.8-3.0 cm in diameter, with associated mediastinal adenopathy (Figure 1).

**Figure 2:**
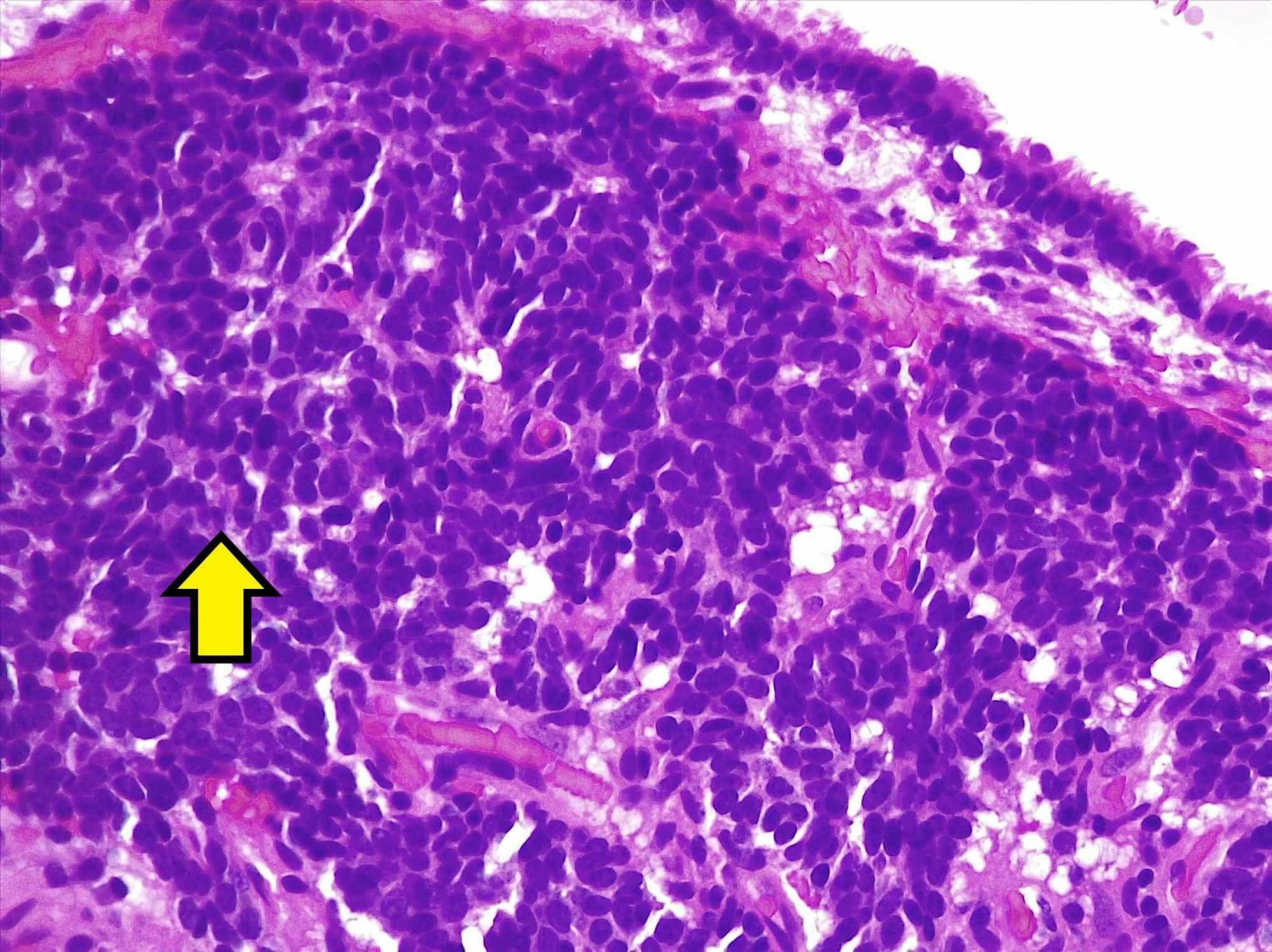
Carcinoid tumor lung, bronchial biopsy (hematoxylin and eosin stain) Pulmonary carcinoid tumor showing solid nests of cells that are relatively large and uniform (yellow arrow)

The patient underwent a left medial assisted thoracic surgery, left upper lobectomy, and mediastinal lymph node dissection. Pathology for the left upper lobe nodule was consistent with a low-grade neuroendocrine carcinoma (typical carcinoid), which was 3.0 cm in diameter, stage pathological stage pT1bN0. Immunohistochemical stains on the tumor revealed large atypical cells, showing positive staining with pancytokeratin, CD56, synaptophysin, and chromogranin, and negative for TTF-1 and cdx-2 (Figure 2).

**Figure 3:**
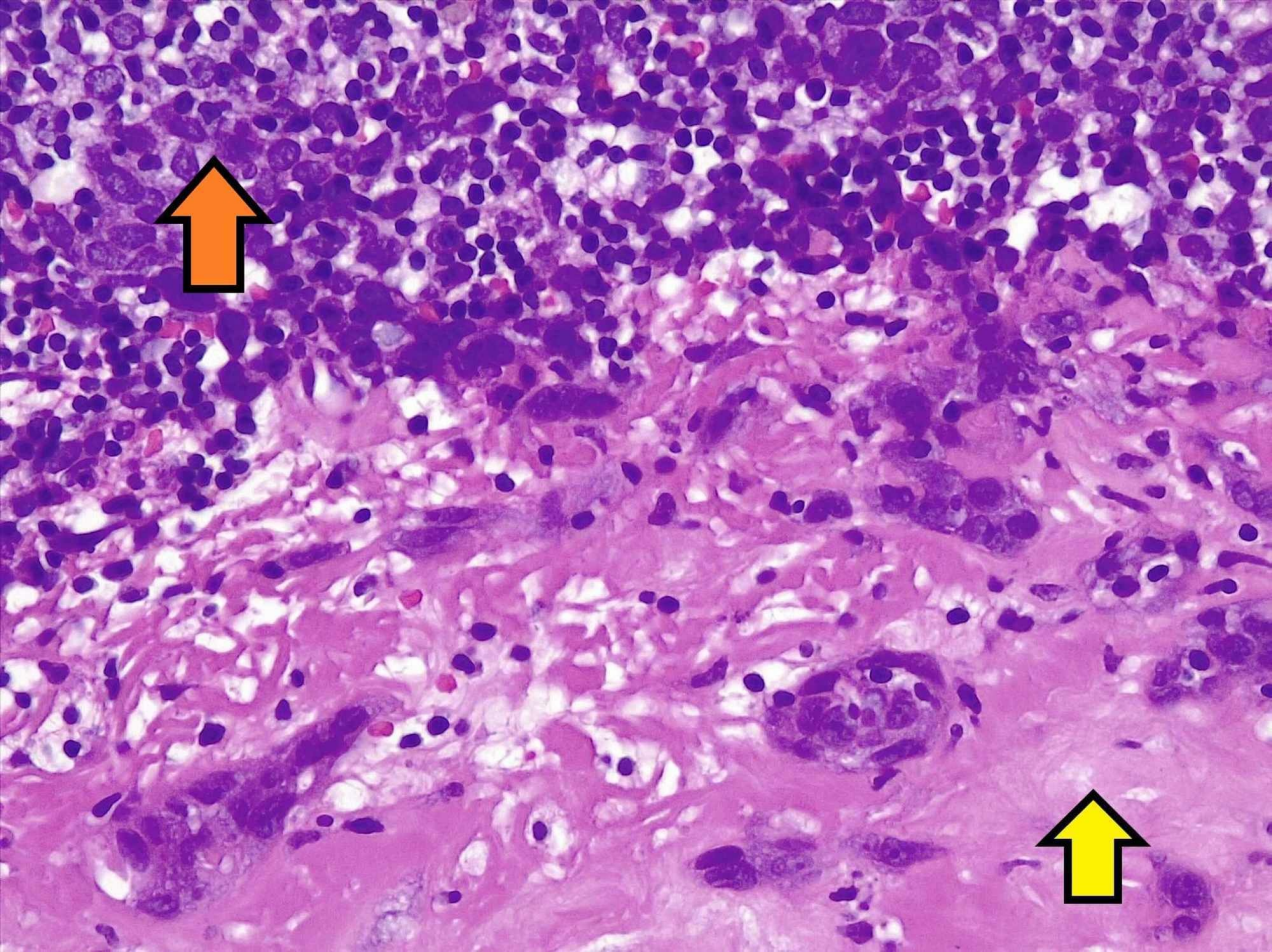
Pathological histology of squamous cell carcinoma, lymph node biopsy (hematoxylin and eosin stain) Large polygonal cells of the squamous cell carcinoma (orange yellow) within an intracellular stroma (yellow arrow)

There was no lymphovascular invasion identified. Bronchial and vascular margins did not reveal any tumor. The seven other lymph nodes that were resected did not reveal a carcinoid tumor. However, one of the level 5 lymph nodes tested positive for metastatic squamous cell carcinoma, non-keratinizing in nature (Figure 3). 

Morphologically, the cells were large and atypical, with immunohistochemical stains staining positive for pancytokeratin and p40. The cells were negative for CK7, Ck20, p16, and TTF-1, suggesting the possibility of a metastatic lesion from an occult lung primary. The carcinoma was immunohistochemically dissimilar from the neuroendocrine tumor. 

A staging positron emission tomography/computed tomography (PET-CT) demonstrated recent postsurgical changes and multiple small pulmonary nodules with non-specific low-level metabolic activity (standardized uptake value [SUV] max =1.8]. There were no evidence of fluorodeoxyglucose (FDG)-avid locoregional or distant sites. Brain MRI showed no evidence of brain metastasis. 

**Figure 4:**
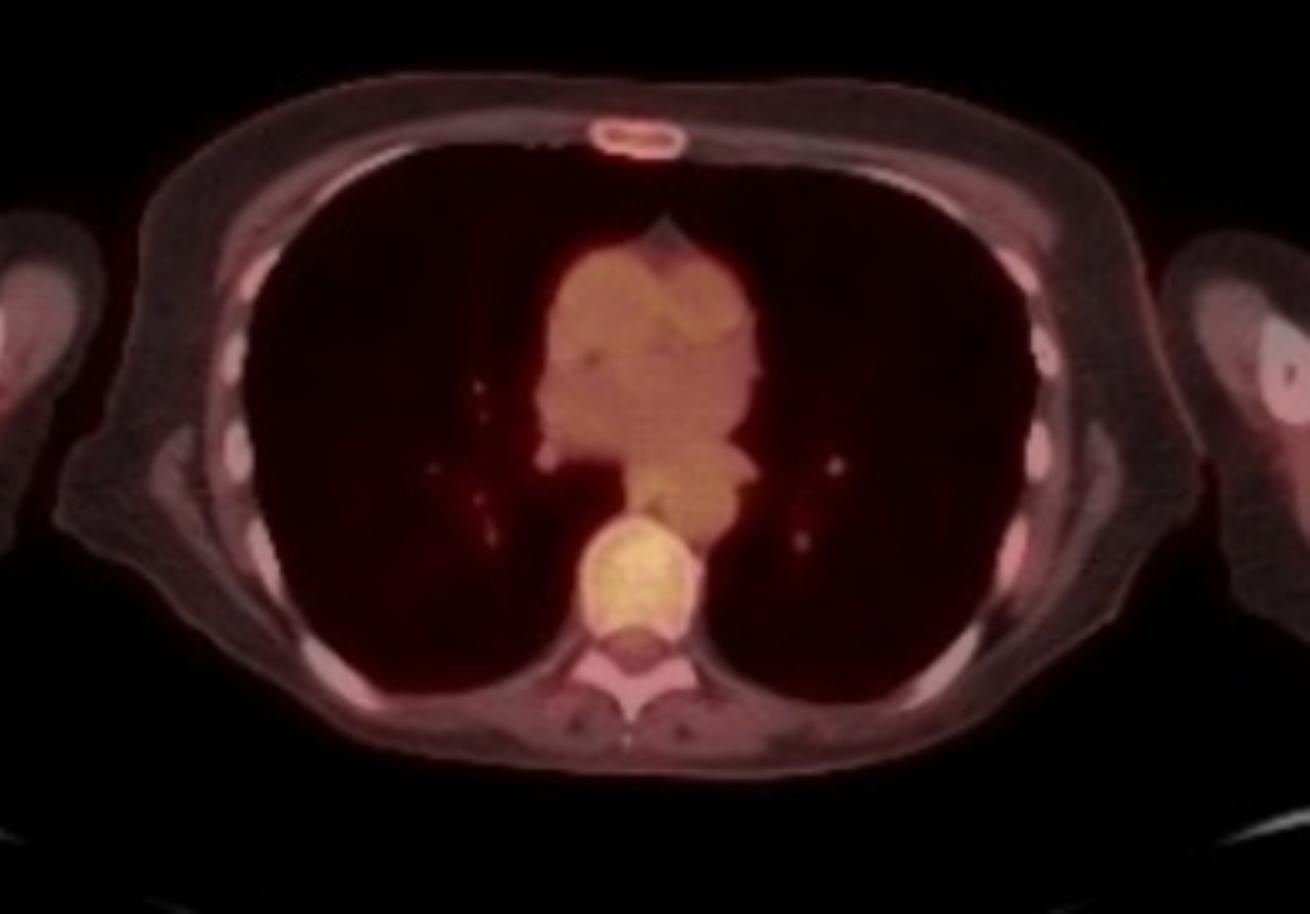
Positron emission tomography/CT (PET-CT) Subsequent and regular PET-CTs have failed to show recurrence or the development of new lesions.

The patient was subsequently treated with postoperative adjuvant chemotherapy with two cycles of cisplatin 70 mg/m^2^ and gemcitabine 1,250 mg/m^2^. However, she developed toxicity with hearing loss and extensive superficial and deep vein thrombosis in the left cephalic, basilic, internal jugular, and left subclavian vein, after which further treatment was halted. The patient subsequently underwent strict surveillance over the next one year with PET-CT scans every three months. Over this span, four scans failed to show new lesions (Figure 4). 

## Discussion

Lung tumors with neuroendocrine differentiation form a group of histologically distinct tumors with varying clinical behaviors ranging from low-grade tumors (typical carcinoid) to intermediate-grade tumors (atypical carcinoid) to high-grade tumors with poor prognosis (large cell neuroendocrine and small cell carcinoma). Pulmonary carcinoid tumors account for about 1%-2% of all lung tumors [2]. To date, there are only a handful of reported cases of synchronous lung tumors containing NSCLC and carcinoid tumors in the medical literature. In one, the authors described a case of combined lung tumor that contained both squamous cell carcinoma and typical carcinoid tumor [3]. Sen and and Borczuk and Saladi et al. documented synchronous lung tumors that comprised both adenocarcinoma and typical carcinoid tumors [1,4]. Furthermore, Yamagata et al. described a case of a laryngeal tumor consisting of both squamous cell and atypical carcinoid tumor components [5]. Okazaki et al. described the case of an NSCLC and atypical carcinoid lung cancer [6]. Synchronous tumors, compared to metachronous tumors, are significantly rarer, with an incidence of only 0.5% of all lung cancers [7].

The pathogenesis of such synchronous tumors is largely unknown. Various studies have proposed the idea of “field” changes in the lung mucosa, giving rise to multiple foci of carcinoma in situ. Slaughter et al. described this phenomenon of “field cancerization” in the aerodigestive tract of smokers, giving rise to squamous cell cancers of the oral cavity. This concept was then extended to include the lung, with the carcinogens found in tobacco damaging the lung mucosa, with a single clonal event resulting in a tumor that subsequently spreads within one or both lungs [8].

Apart from differentiating typical carcinoids from atypical carcinoids, a distinction must be made between synchronous primary tumors and intrapulmonary metastases. As mentioned above, determining the type of these tumors will dictate subsequent treatment approaches. However, this differentiation can prove to be challenging. With the recent advances in imaging, genetic, molecular, and pathological techniques, the differentiation of synchronous tumors from metastatic tumors has become easier.

The optimal management of these patients with multiple primary tumors is controversial. As a result, since combined lung tumors that contain both NSCLC and carcinoid tumors are even rarer, their prognosis is unclear. Most reports agree that surgical resection, if feasible, is the treatment of choice and does not need any further systemic or adjuvant therapy. In a retrospective study conducted by Finley et al, 175 patients underwent surgical resection for synchronous tumors. A total of 25 patients (14%) were given adjuvant therapy: 17 chemotherapy and 8 external beam radiotherapy [9]. In contrast, in another retrospective study, survival was analyzed in 26 patients who had undergone surgical resection for multiple synchronous non-small-cell lung tumors. The five-year survival rate was 49.7%, with a median survival time of 40 months. They also found that the three-year survival rate was 66.7% for patients who received adjuvant chemotherapy and/or radiotherapy, compared to others (56.3%) [10]. In both studies, given the limited study population, it is difficult to conclude the value of adjuvant therapy. Both studies excluded patients with synchronous carcinoid tumors. Currently, surgery remains the only curative option for both typical and atypical carcinoids. The RAD001 in Advanced Neuroendocrine Tumors, Fourth Trial (RADIANT4) was a phase III trial that found a reduced incidence of disease progression and death with everolimus compared with placebo in patients with bronchial neuroendocrine tumors (NETs) [11]. Such treatment in patients with both carcinoid and tumor of another histological type is not studied. Our patient underwent surgical resection of both pulmonary tumors and only two cycles of chemotherapy and has remained disease-free since then. In the report by Okazaki et al., the patient, despite undergoing surgical resection of the atypical carcinoid and NSCLC along with postoperative adjuvant chemotherapy, developed widespread disease progression to the brain and adrenal glands that was resistant to subsequent chemotherapy and radiotherapy [6]. Studies have shown that patients with an atypical carcinoid have a five-year survival rate that is between 60% and 80%, while those with typical carcinoids have a similar five-year survival rate that is 90%-100% [12].

## Conclusions

Our patient had a rare association between squamous cell lung cancer and bronchial lung carcinoid. In addition to increased awareness and rapid diagnosis, additional sufficiently powered controlled clinical trials of patients with synchronous lung carcinoids are needed to improve evidence-based care and make recommendations that outline optimal local or systemic treatment. All synchronous or metachronous tumors must be investigated and not assumed to be intrapulmonary metastasis, as early-stage tumors may be prone to surgical resection. Strict postoperative surveillance with history, physical exam, and imaging studies is mandatory to monitor for disease recurrence.
